# The Computerized Cognitive Composite (C3) in A4, an Alzheimer’s Disease Secondary Prevention Trial

**DOI:** 10.14283/jpad.2020.38

**Published:** 2021

**Authors:** K.V. Papp, D.M. Rentz, P. Maruff, C.-K. Sun, R. Raman, M.C. Donohue, A. Schembri, C. Stark, M.A. Yassa, A.M. Wessels, R. Yaari, K.C. Holdridge, P.S. Aisen, R.A. Sperling

**Affiliations:** 1 Department of Neurology, Brigham and Women’s Hospital and Harvard Medical School, Boston, Massachusetts, USA; 2 Department of Neurology, Massachusetts General Hospital and Harvard Medical School, Boston, MA, USA; 3 The Florey Institute of Neuroscience and Mental Health, University of Melbourne, Parkville, Victoria, Australia; 4 Cogstate, Ltd, Melbourne, Victoria, Australia; 5 Alzheimer Therapeutic Research Institute, Keck School of Medicine, University of Southern California, San Diego, CA, USA; 6 Center for the Neurobiology of Learning and Memory and Department of Neurobiology and Behavior, University of California Irvine, Irvine, California, USA; 7 Eli Lilly and Company, Indianapolis, Indiana, USA

**Keywords:** Digital biomarkers, cognition, computerized testing, preclinical Alzheimer’s disease, secondary prevention

## Abstract

**BACKGROUND:**

Computerized cognitive assessments may improve Alzheimer’s disease (AD) secondary prevention trial efficiency and accuracy. However, they require validation against standard outcomes and relevant biomarkers.

**OBJECTIVE:**

To assess the feasibility and validity of the tablet-based Computerized Cognitive Composite (C3).

**DESIGN:**

Cross-sectional analysis of cognitive screening data from the A4 study (Anti-Amyloid in Asymptomatic AD).

**SETTING:**

Multi-center international study.

**PARTICIPANTS:**

Clinically normal (CN) older adults (65–85; n=4486)

**MEASUREMENTS:**

Participants underwent florbetapir-Positron Emission Tomography for Aβ+/− classification. They completed the C3 and standard paper and pencil measures included in the Preclinical Alzheimer’s Cognitive Composite (PACC). The C3 combines memory measures sensitive to change over time (Cogstate Brief Battery-One Card Learning) and measures shown to be declining early in AD including pattern separation (Behavioral Pattern Separation Test- Object-Lure Discrimination Index) and associative memory (Face Name Associative Memory Exam- Face-Name Matching). C3 acceptability and completion rates were assessed using qualitative and quantitative methods. C3 performance was explored in relation to Aβ+/− groups (n=1323/3163) and PACC. RESULTS: C3 was feasible for CN older adults to complete. Rates of incomplete or invalid administrations were extremely low, even in the bottom quartile of cognitive performers (PACC). C3 was moderately correlated with PACC (r=0.39). Aβ+ performed worse on C3 compared with Aβ− [unadjusted Cohen’s d=−0.22 (95%CI: −0.31,−0.13) p<0.001] and at a magnitude comparable to the PACC [d=−0.32 (95%CI: −0.41,−0.23) p<0.001]. Better C3 performance was observed in younger, more educated, and female participants.

**CONCLUSIONS:**

These findings provide support for both the feasibility and validity of C3 and computerized cognitive outcomes more generally in AD secondary prevention trials.

## Introduction

Computerized cognitive assessments have the potential to significantly reduce data administration and scoring errors, site burden, and cost in Alzheimer’s disease (AD) secondary prevention trials as cognitive screening tools and outcome measures. These assessments have yet to replace paper and pencil measures as primary outcomes given several remaining questions: How feasible are computerized assessments in normal older adults and older adults who progress to Mild Cognitive Impairment (MCI) over the course of a trial? How reliable is the data collected? And finally, how valid are computerized cognitive assessments, that is, are they related to gold-standard paper and pencil primary outcomes and AD pathology targeted in a given intervention?

The Anti-Amyloid in Asymptomatic Alzheimer’s (A4) study (1, 2) offers a unique opportunity to address some of these questions by assessing the feasibility and validity of the Computerized Cognitive Composite (C3) in a very large multi-site AD secondary prevention study targeting clinically normal (CN) older adults with elevated cerebral amyloid (2). The C3 is derived using two well-validated memory paradigms from the cognitive neuroscience literature: the Face Name Associative Memory Exam (FNAME) and the Behavioral Pattern Separation Task-Object (BPS-O). It also includes measures from the Cogstate Brief Battery (CBB) which uses playing cards to assess visual memory in addition to reaction time (RT) and working memory and was designed to be sensitive to change over time with randomized alternate forms. The CBB has been studied in relationship to AD neuroimaging markers in several cohort studies of normal older adults (3, 4). Behavioral versions of the FNAME (5, 6) and a modified version of the BPS-O (7) were selected for inclusion in the C3 as they have been shown to elicit aberrant activity in the medial temporal lobes during functional imaging studies in individuals at risk for AD based on biomarkers (8–10). More specifically, these individuals fail to habituate to repeated stimuli (FNAME) or during both correct rejections and false alarms (BPS-O), neural signatures consonant with successful memory formation. The C3 was identified a-priori to include one primary memory outcome from each component measure including: the BPS-O lure discrimination index, Face-Name Matching accuracy, and One-Card Learning accuracy.

The aim of this study was to assess the feasibility and validity of the C3 in CN older adults participating in a secondary prevention trial. Specific goals included determining whether reliable C3 data was consistently captured using a touchscreen tablet and whether data reliability decreased in the lowest cognitive performers. To assess the validity of the C3, we investigated 1) whether the C3 was related to the primary study outcome: performance on traditional paper and pencil measures (i.e., the Preclinical Alzheimer’s Cognitive Composite- PACC) 2) whether the C3 was related to cerebral amyloid (Aβ) and 3) whether the magnitude of this relationship was comparable to that observed between PACC and Aβ+/−. In addition to our main aims, we explored whether improved performance with C3 retesting using alternate forms differentiated between Aβ+/− individuals above and beyond cross-sectional performance. Finally, we explored performance on the constituent tests from the C3 and their relationships with Aβ status, demographic characteristics, and paper and pencil measures. The implications of these findings as they relate to the design and use of future computerized outcomes in secondary prevention trials are discussed.

## Methods

### Participants and Study Design

The A4 Study is a double-blind, placebo-controlled 240-week Phase 3 trial of an anti-Aβ monoclonal antibody in CN older adults with preclinical AD (2) occurring across 67 sites. Participants interested in enrolling in A4 were required to be aged 65 to 85 and were deemed clinically normal (CN) based on Mini Mental Status Exam (MMSE) ranging from 25–30 and Global Clinical Dementia (CDR) Rating Score of 0. During their initial screening visit, participants completed traditional and computerized cognitive testing (detailed further below). Prior to enrollment, they underwent a florbetapir Positron Emission Tomography (PET) for classification of Aβ status (Table 1) at a second visit. On their third visit, all potential participants completed computerized testing and were subsequently provided with results of their AD biomarker imaging and informed about whether they were eligible (Aβ+) or ineligible (Aβ−) to enroll in the trial. The current study includes cognitive screening data at 2 timepoints for Aβ+ and Aβ− individuals.

### Cognitive Measures

The primary outcome for the A4 Study is performance on the PACC, a multi-domain composite of paper and pencil measures (11). Measures contributing to the C3 are administered on a touchscreen tablet using the Cogstate platform and serve as an exploratory outcome. All participants completed the PACC and C3 at the first screening visit (Visit 1) and an alternate C3 within 90 days (mean=55 days) at the study eligibility visit (Visit 3) prior to study eligibility disclosure.

### Paper and Pencil Cognitive Testing: The PACC

The PACC, described in detail elsewhere (11), is calculated as the sum of mean performance across four measures normalized using a z-score including the MMSE (0–30), the WMS-R Logical Memory Delayed Recall (LMDR; 0–25), the Digit-Symbol Coding Test (DSC; 0–93), and the Free and Cued Selective Reminding Test–Free + Total Recall (FCSRT96; 0–96) (2).

### Computerized Testing: The C3

**Figure 1.**
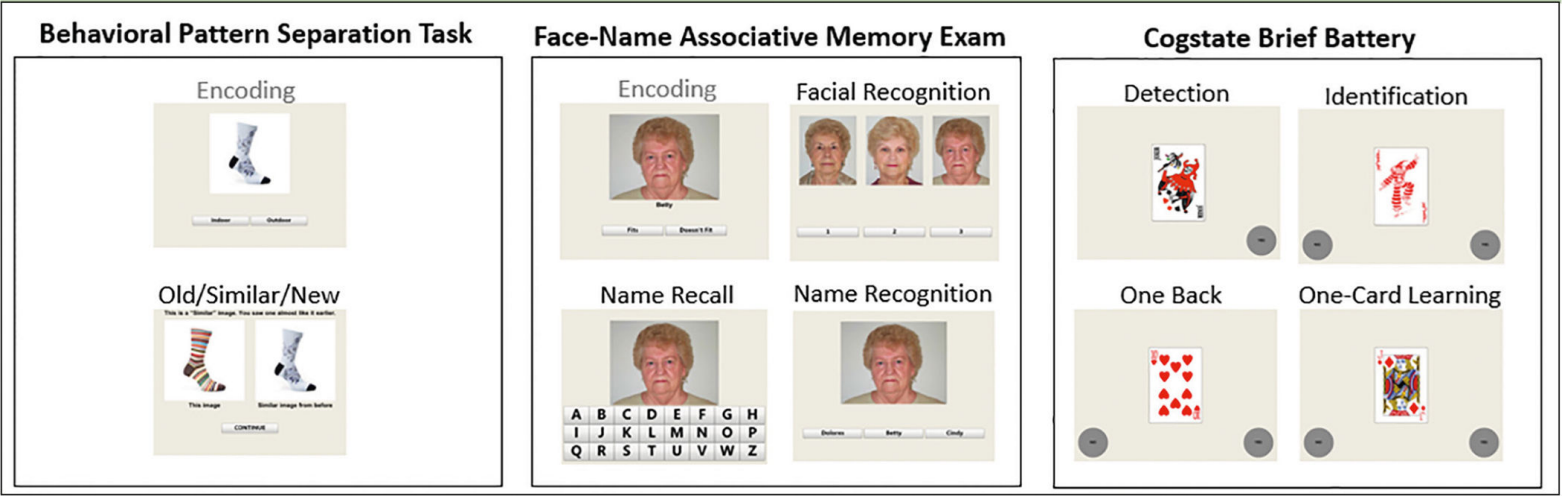
C3 Task Schematic Note. All tasks are completed on a tablet using a touchscreen. Stimuli in gray are not scored.

Figure 1 provides a schematic of C3 Components: BPS-O, FNAME and the CBB. An examiner is present in the testing room and initially guides administration, but the battery has the potential to be completed largely independently in the context of written on-screen instructions and automatic transitions between tasks (12).

### Behavioral Pattern Separation- Object (BPS-O; more recently termed the Mnemonic Similarity Test)

Participants are presented with images of 40 everyday objects serially and are allotted 5 seconds to determine whether the item is for use “indoors” or “outdoors” to ensure adequate attentiveness to stimuli (7). Participants are subsequently shown 20 of the same items interspersed with both novel images and lure images. They are asked to categorize each image as: Old, Similar, or New within 5 seconds. Accuracy and RT measures are collected. Of interest is the rate at which participants can correctly identify lures as “Similar” rather than as “Old.” The lure discrimination index (LDI) is computed as the proportion of “Similar” responses given to lure items minus the ratio of “Similar” responses given to the foils (the latter is to correct for response bias). The LDI is the primary outcome from the BPS-O task. A higher LDI indicates better pattern separation performance.

### Face-Name Associative Memory Exam (FNAME)

Participants are shown 12 face-name pairs presented serially. For each face-name pair, the participant is asked whether the name “fits” or “doesn’t fit” the face to ensure adequate attentiveness to the stimuli. Participants are allowed 5 seconds to respond and are asked to try to remember the face-name pair. Following the learning phase, the CBB tests serve as a 12 to 15-minute delay. Subsequently, there are three measures of memory including face recognition (FSBT), first letter name recall (FNLT) and face-name matching (FNMT). In FSBT, participants are asked to identify the previously learned faces, presented alongside two distractor faces of matching age, race, and sex. The target face is subsequently presented with a touchscreen keyboard and the participant selects the first letter of the name paired with that face (FNLT). Finally, the target face is presented with three names (target name, a re-paired same-sex name, and an age and sex-matched foil name) and the participant must select the correct name (FNMT). Accuracy for each component is scored /12 with FNMT number of correct matches serving as the primary outcome of interest.

### Cogstate Brief Battery (CBB)

The CBB (13, 14) uses playing cards as stimuli and includes a measure of attention (Detection-DET),reaction time RT (Identification-IDN), working memory (One-Back Test-ONB), and visual memory (One-Card Learning-OCL). Measures of RT and accuracy are recorded. To address skewness, a log10 transformation is applied to RT measures and an arcsin sqrt transformation is applied to accuracy measures. In DET, participants are required to tap ‘Yes’ as quickly as possible in response to a stimulus card turning face-up. The task continues until 35 correct trials are recorded. The outcome is RT. In IDN, a participant must select whether the card is red or not red; thirty correct trials are required. RT is the primary outcome for IDN; IDN accuracy was also examined. In ONB, participants must indicate “yes” or “no” whether the current card is equivalent to the previously seen card. In OCL, participants must learn a series of playing cards by responding ‘yes’ or ‘no’ to whether the card has been previously seen in the task. For ONB and OCL, both RT and accuracy are computed. Here, we examined RT and Accuracy for both IDN and ONB. We examined only RT for DET and only Accuracy for OCL.

### The C3

Constituents of the C3 were identified a-priori and include one primary memory outcome from each measure including the BPS-O LDI, FNMT, and OCL. The C3 is computed as the average of these z-scored outcomes derived from the study population at Visit 1.

### Data Quality

Data from individual C3 measures were included in analyses if they met pre-specified task-specific completion checks (Supplementary Table 1). For example, OCL for a given participant is included in analyses if the participant responds in ≥75% of trials. Study rater comments were also reviewed to better determine C3 usability and acceptability.

### Amyloid PET Imaging

Eligible participants completed a florbetapir PET scan at Visit 2. Scan acquisition occurred over 50–70 minutes following an injection of 10mCi of florbetapir-F18. Aβ binding was assessed using mean standardized uptake value ratio (SUVr) with whole cerebellar gray as a reference region. Participants were deemed eligible (Aβ+) versus not eligible (Aβ−) using an algorithm combining both quantitative SUVr (>1.15) information and a centrally-determined visual read (2).

### Statistical Analyses

Primary analyses were performed on the C3 at Visit 1. To assess C3 feasibility and data validity, test completion rates and performance checks were computed (Supplementary Table 1) and rates subsequently compared between Aβ+/− groups using Chi-square tests. Rater comments were systematically reviewed and observations by raters were grouped into categories (e.g., technical issue, interruptions) and the frequency of observations made in each category were computed. To infer C3 feasibility and data validity in those who may develop impairment over the course of the A4 study, we compared test completion rates and performance checks between the lowest cognitive performers (bottom quartile on PACC) with typical cognitive performers using *chi* square tests.

Demographic differences between Aβ+/− groups were assessed using Welch’s two-sample t-tests for continuous variables and Fisher’s Exact test for categorical variables (e.g., age, APOE). Linear models were fit to compare cognitive performance across males and females. Linear models were fit to compare cognitive performance across Aβ+/− while adjusting for covariates: age, sex, and education. Effect size was computed as a Cohen’s d (mean difference between Aβ+ and Aβ− groups divided by the pooled standard deviation) with 0.01 representing a “very small” effect, 0.20 representing a “small” effect, and 0.5 representing a “medium” effect (15). Comparable linear models were performed and effect sizes calculated for individual C3 components to examine Aβ+/− group differences on individual C3 measures (e.g., OCL, ONB, BPS-O). No adjustments were made for multiple comparisons; however, results are reported as point estimates and 95% confidence intervals.

Differences in performance between Visit 1 and Visit 3 were examined using linear models of difference scores with Aβ status, age, sex, and education as covariates.

Pearson correlation coefficients were computed to assess the relationships between C3 and demographic characteristics as well as C3 and the PACC. Pearson correlation coefficients were similarly used to assess the relationships among C3 components and PACC components to assess the convergent and discriminant validity between memory versus non-memory tasks on C3 versus PACC.

Linear models were also fit to compare cognitive performance between ε4+/− while adjusting for covariates: age, sex, and education.

All analyses were conducted using R version 3.6.1 (R-project.org).

## Results

### Feasibility of the C3

Completion and performance checks were met in >98% of individual test administrations within the C3 (Supplementary Table 1) and equivalent by Aβ+/. Raters reported issues in approximately 4% of C3 administrations. The most commonly reported problem (reflecting 0.7% of administrations) was that the tablet was insufficiently responsive to a participant’s finger taps and/or the participant was mis-tapping by either hovering their fingers too closely to the screen or by tapping too quickly. The second most commonly reported issue (0.5% of administrations) was overly deliberative responding on BPS-O and FNAME causing items to time-out. This was followed by non-specific technical issues (e.g., frozen program, interruptions from low battery signal or software update, glitches such as stimulus not loading or items auto-proceeding). Report of confusion with task instructions was very low (reported in 0.3% of administrations). Participants most commonly had difficulty understanding instructions for ONB and OCL; additionally, some reported confusion regarding the goal of the judgment component of BPS-O and FNAME learning components (i.e., indoor vs. outdoor, fits vs. doesn’t fit). Despite this, few participants (<3%) failed to make an “indoor/outdoor” or a “fits” judgment on more than 3 items. Participants refused to continue C3 testing in <0.002% of administrations with the most common reasons including frustration and fatigue.

### Predictions for the Feasibility of the C3 Longitudinally

To preliminarily estimate whether the C3 (to be completed at 6-month intervals for the A4 study duration) will remain feasible in participants experiencing cognitive decline, we examined C3 performance in the lowest cognitive performers on PACC. The magnitude of the C3 Aβ group difference increased by a factor of 5.2 when restricting the Aβ+ group to the bottom quartile of PACC [adjusted cohen’s d=−0.57 (95%CI:−0.68, −0.45) p<0.001], however, no significant changes in rates of performance completion and performance checks were observed.

### Demographic and Clinical Characteristics

Aβ+ were older compared with Aβ− (Table 1). There were no group differences for sex or education level. Aβ+ exhibited a higher rate of ε4 positivity and higher proportion of Caucasians compared with Aβ−.

### C3 Performance

**Figure 2.**
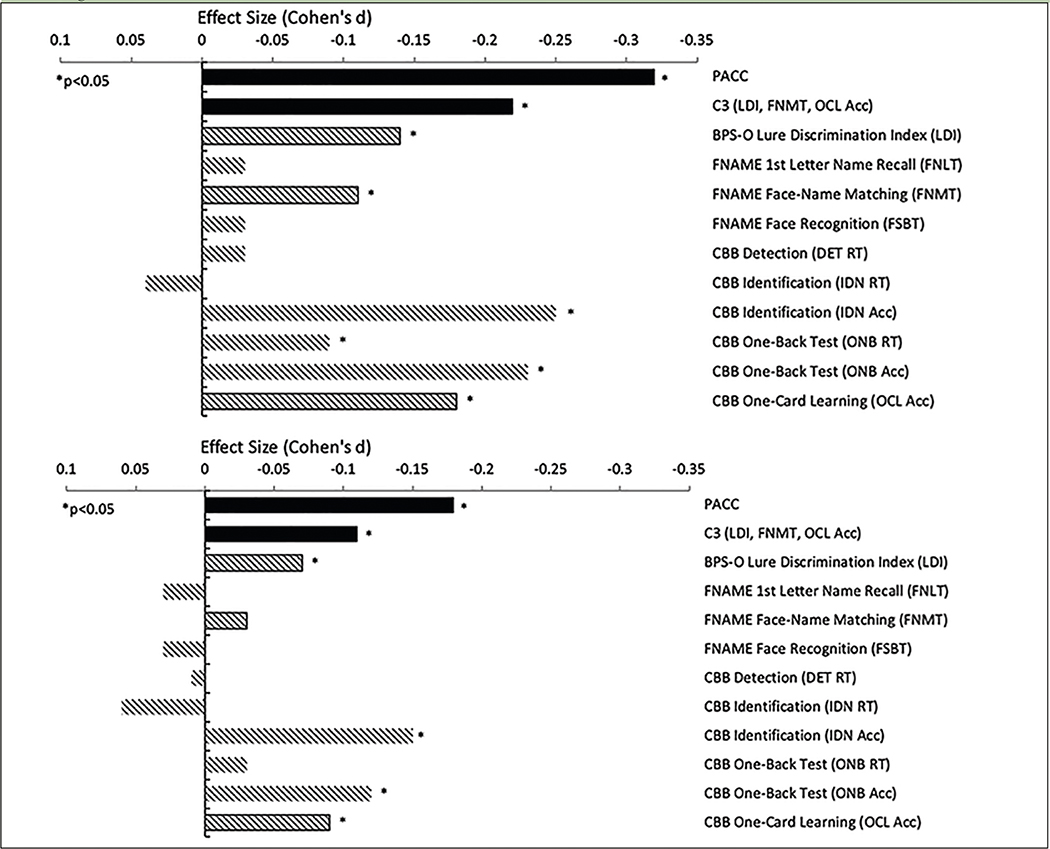
Covariate-Unadjusted and Adjusted Group Differences (Effect Sizes: Cohen’s d) Between Aβ+/Aβ− Groups at Screening Visit 1 Note. Smaller effect size (Cohen’s d) is associated with worse performance in Aβ+ (n=1323) relative to Aβ− (n=3163). Top (unadjusted) and bottom (covariate-adjusted). PACC=Preclinical Alzheimer’s Cognitive Composite; C3= Computerized Cognitive Composite; FNAME=Face-Name Associative Memory Exam; CBB=Cogstate Brief Battery; RT=reaction time; Acc=Accuracy

Aβ+ performed worse on the C3 compared with Aβ− (unadjusted d=−0.22, adjusted d=−0.11), mirroring the Aβ+/− performance difference on the PACC (unadjusted d=−0.32, adjusted d=−0.18) (Figure 2; Table 2). Importantly, the majority of participants were performing in the normal range, with performance in Aβ+ on average only −0.08 standard deviations below the mean. In addition to Aβ positivity (Beta=−0.07 p=0.002), older age (Beta= −0.04 p<0.0001), less education (Beta= 0.03 p<0.0001), and male sex (Beta=−0.10 p<0.0001) contributed to overall worse C3 performance. Models adjusted for demographic features generally resulted in smaller Aβ+/− effect sizes compared with unadjusted models (Figure 2). For example, there was 66% decrease in effect size between the unadjusted (d=−0.22) and adjusted C3 (d=−0.11). C3 and PACC were moderately correlated (r=0.39, p<0.001). However, both contributed unique explanatory variance about Aβ+/− status when modeled together (Supplementary Table 2 Model A).

Improved performance at re-testing was observed for C3 with an average increase of 0.25 standard deviations between visits (Beta=0.25, p<0.0001). However, there was no relationship between Aβ status and differential improvement on C3 re-testing (Beta= 0.00, p=0.961). Importantly, Aβ+ continued to perform worse on the C3 compared with Aβ− and this group difference was at a comparable magnitude as compared with initial testing (re-testing cohen’s d=−0.21, p<0.0001).

### Individual C3 Components

Individual C3 components which showed statistically significant differences between groups were BPS-O LDI, FNAME FNMT, CBB IDN accuracy, ONB accuracy and RT, and OCL accuracy. When adjusting for demographics, FNAME FNMT and ONB RT were no longer significant. Interestingly, for IDN RT, Aβ+ exhibited a statistical trend towards unexpectedly faster RT compared with Aβ− (adjusted d=−0.06, p=0.055). Despite a trend towards being slightly faster, Aβ+ were less accurate for IDN compared with Aβ− (unadjusted d=−0.25, adjusted d=−0.14). IDN Accuracy was correlated with IDN RT (r= −0.30, p<0.001) such that generally faster RT for correct responses was associated with reduced overall accuracy. However, when both IDN Accuracy and IDN RT were incorporated into the sample model to predict Aβ status, only reduced IDN Accuracy was a significant predictor (Supplementary Table 2 Model B).

### Correlations Among C3 Components, Demographics, PACC

#### Age

Greater age was associated with worse performance across all C3 outcomes (Table 3). This association was strongest for the overall C3 Composite (r=−0.29, p<0.001). Age was least associated with RT tasks including DET (r=−0.13, p<0.001) and IDN (r=−0.11, p<0.001).

#### Education

Higher education was associated with better performance on all individual C3 outcomes, with the largest impact on OCL accuracy (r= 0.13, p<0.001) followed by the overall C3 (r=0.12, p<0.001). The only exception was ONB RT where faster performance was associated with lower education.

#### Sex

Women outperformed men on all components of FNAME including FNLT (d= −0.46, p<0.0001), FNMT (d= −0.36, p<0.0001), and FSBT (d= −0.39, p<0.0001). Women also outperformed men on IDN Accuracy (d= −0.16, p<0.0001) and ONB Accuracy (d=−0.08, p=0.019). Interestingly, however, men outperformed women on DET (d= −0.23, p<0.0001) and ONB RT (d= −0.12, p<0.001). Performance between the sexes was comparable for BPS-O, IDN RT, and OCL Accuracy.

On OCL, Aβ+ females did not perform differently compared with Aβ− females [Estimate=−0.00 (0.01), p=0.468]. However, Aβ− males performed worse compared with Aβ+ males [Estimate=−0.02 (0.01), p=0.0006]. This suggests that OCL captures subtle decrements in memory between Aβ+/− men but not women. A non-significant statistical trend toward the same pattern was observed in BPS-O.

#### PACC and C3

Correlations among components of the 2 composites tended to be more strongly-related in a domain-specific manner providing support for convergent and discriminant validity (Table 3). For example, DET and IDN were correlated with DSST at r=0.26 and 0.31, respectively while not being significantly related to memory components of the PACC (FCSRT, Story Memory) or MMSE.

### The C3 and APOE Status

There was no difference in performance between APOEε4 carriers vs. non-carriers on the C3 [adjusted d= −0.03 (95% CI: −0.09, 0.03), p=0.379] or on individual C3 outcomes (not shown). The model for carrier vs. non-carrier group differences did not improve with the removal of demographic covariates in contrast with models for Aβ+/− [unadjusted d= 0.03 (95% CI: −0.05, 0.10), p=0.470]. Finally, we did not observe an interaction between E4 and Aβ status on the C3.

## Discussion

Among a large sample of CN older adults screening for an AD secondary prevention trial, assessment of cognition using a tablet-based measure (C3) was feasible. Diminished C3 performance was associated with worse PACC performance and elevated Aβ. Although the magnitude of the Aβ+/− group difference was statistically small (d= −0.11, once adjusted for covariates) it was comparable to that observed on well-established and clinically meaningful paper and pencil measures included in the primary outcome, i.e., the PACC (d= −0.18). Performance on the C3 was also reliable, with an equal Aβ+/− group effect on the C3 at retesting within 90 days. More broadly, these findings suggest that computerized testing has the potential to replace traditional paper and pencil primary outcomes in future trials- representing a potential shift in clinical trial cognitive assessment methodology. Additionally, these results further confirm the small but consistent association between Aβ burden and cognition cross-sectionally within a CN population.

### Usability/Acceptability of the C3

The very low rates of incomplete and/or invalid administrations for the C3 battery indicate that in the older adults assessed, even those with little computer literacy, the supervised tablet-based cognitive testing has high acceptability. Rates of completion and performance check failures remained low in a subset of low PACC performers, providing early evidence for C3 feasibility longitudinally as some participants show progressive cognitive decrements over the course of the study. Study procedures required a rater to supervise C3 testing, however, raters noted that many participants did not require significant assistance after completing the first few measures. This was further evidence by improved performance on re-testing as participants gained familiarity with the device and tasks. Future trials may consider further optimizing computerized tasks to be self-guided to reduce rater training and time. Potential barriers to tablet-based testing were infrequent, largely addressable, and unlikely to systematically affect performance on the C3. These included inexperience with tablets leading both to mis-tapping and difficulty registering finger taps. Many older adults emphasized accuracy over speed during learning trials, resulting in time-outs. Several of these issues can be addressed with modifications to instructions and design (e.g., including a timer indicator) while others will diminish over time with secular trends toward increased familiarity with digital technology.

### The C3 Composite and Individual C3 Measures by Aβ+/−

Components of C3 tests which differed between Aβ+/− groups were primarily in memory (BPS-O; OCL) but also included working memory (ONB). The difference in pattern separation memory performance between Aβ+/− participants extends previous fMRI works showing an association between AD biomarkers (including Aβ -PET) and aberrant fMRI activity during learning on a pattern separation task in normal older adults (9) to a difference in frank performance. The BPS-O (10) was designed in part to capture a weakened “novelty signal”, that is, a reduced ability to correctly discriminate between stimuli that are similar but not identical to previously encountered targets. This tendency to misidentify similar lures as targets has been conceptualized as an error in pattern separation (16). Aβ group differences were also observed on face-name memory but this effect was significantly attenuated when controlling for demographic features. In contrast with other C3 memory measures (OCL Accuracy and BPS-O) there was a significant sex effect whereby women generally performed better on all aspects of FNAME compared with men. This may be attributable to a general female advantage in verbal memory (17), however, it may be related to the nature of the information. Previous work with FNAME indicates a diminishment of the sex effect when requiring memory for occupation-face versus name-face pairs (5, 18). Our findings from the CBB measures were consistent with previous results examining this battery in relationship to AD neuroimaging markers in normal older adults. Poorer performance on OCL has been associated with higher levels of CSF phosphorylated-tau/Abeta42 in late middle-aged participants in the Wisconsin Registry for Alzheimer’s Prevention (4). Similarly, we found that OCL was sensitive. However, we also found that working memory (ONB) was also relatively strongly associated with elevated Aβ. While C3 constituents were selected theoretically and a-priori, ONB may be considered for inclusion in future optimized and/or data-driven C3 versions. Interestingly, the Aβ+ group made more errors on a Cogstate RT task (IDN) but paradoxically also performed the task more quickly compared with the Aβ− group. These findings suggest that faster RT may, in fact, be a sign of subtle decrements. One explanation for this finding is an age-associated decrease in inhibition of pre-potent responses (19) may be more pronounced in preclinical AD. More broadly, it confirms that early cognitive changes in preclinical AD extend beyond memory (20, 21).

Part of the impetus for combining outcomes from the BPS-O, FNAME, and CBB into a C3, is aligned with the rationale for cognitive composites as primary endpoints (22) to maximize signal to noise ratio in a population expected to exhibit subtle cognitive decrements. This was confirmed in our data whereby the combination of FNMT, BPS-O, and OCL into the C3 resulted in a numerically larger effect size compared with any single one of these measures alone. However, there are multiple means of constructing composites including data-driven approaches; for example, selecting measures most associated with Aβ cross-sectionally or measures most sensitive to change. The current C3 was theoretically derived on the basis of previous literature and longitudinal data is needed to confirm its sensitivity over time. Importantly, different memory measures provided related but partially unique information about Aβ status. For example, both BPS-O and OCL were significant predictors of Aβ status when included in the same model (Supplementary Table 2 Model C). More recent work examining the heterogeneity of cognitive decline in early AD suggests that different atrophy patterns are associated with different cognitive trajectories (23). A cognitive composite would thus benefit from being sufficiently broad to avoid under/overestimating decline in a given subgroup.

Our finding that OCL differentiated Aβ+ vs Aβ− men but not women highlights the issue of heterogeneity in a different light. Males and females performed equivalently for visual memory of playing cards (OCL) but females outperformed males on face-name memory. We hypothesize that visual card-based tasks may be both more engaging and an area of relative strength for males versus females in contrast with name memory (17). Regardless, these findings highlight the rationale for composite scores and the opportunity to use C3 to better understand demographic and individual differences in performance and cognitive trajectories.

### C3 Performance and ε4 Status

The lack of a group difference in C3 performance between ε4 carriers vs. non-carriers is not unexpected given the specific recruitment of CN older adults and the current cross-sectional analysis. This is evidenced by the further diminishment of group differences between e4+ vs. e4− participants when including age as a covariate. In contrast, removal of age as a covariate systematically increased the Aβ+ vs. Aβ− group differences.

### C3 and Re-testing

Consistent with the literature, participants performed slightly better on re-testing which is consistent with increased familiarity with the tablet and task demands (3). Diminished practice effects have been shown to predict incident MCI and/or dementia (24, 25) and have been suggested as a screening tool (26). However, we did not observe differential improvement in performance by Aβ group status. Future adjustments to the FNAME paradigm emphasizing item versus task familiarity may increase the relevance of a diminished practice effect. More specifically, using repeated versus alternate stimuli may capture more AD-specific learning over repeated exposures to the same material (27). C3 practice effects are likely to diminish significantly after the second administration (24). Likewise, item familiarity practice effects are unlikely to contribute to C3 trajectories over time given that all remaining versions are unique.

## Conclusions

Within the context of AD secondary prevention trials, our results indicate that computerized (tablet-based) cognitive testing is feasible in older adults in a secondary prevention trial setting and we provide support for the validity of such testing as the C3 was 1) correlated with the primary outcome of paper and pencil composite performance (PACC), 2) related to AD pathological burden (Aβ+/−) and 3) related to Aβ+/− at a similar magnitude as the PACC. Positive relationships with AD biomarkers and PACC suggest that the C3 is capturing meaningful cognitive decrements and, has the potential to serve as a proxy for paper and pencil measures in future trials. In addition to reducing staff time and allowing the possibility for remote assessment, computerized testing has the potential to capture a greater quantity and more nuanced quality of data for each measure. Future work will determine the sensitivity of the C3 to change over time in the context of an anti-amyloid treatment trial.

## Supplementary Material

Supplementary Material

## Figures and Tables

**Table 1.** Participant Characteristics by Aβ Status

	All n=4486	Aβ-n=3163	Aβ+ n=1323	p-value
Age M(SD)	71.29 (4.67)	70.95 (4.53)	72.10 (4.89)	<0.0001
Sex (% female)	59%	60%	59%	0.641
Education M(SD)	16.58(2.84)	16.59 (2.85)	16.54(2.81)	0.564
APOE Genotype (% ε4+)	35%	25%	58%	<0.001
Race (% Caucasian)	92%	91%	94%	<0.001

Note. Two-sample t-test with unequal variances were used for continuous variables and Fisher’s Exact test for categorical variables. Values are Mean (Standard Deviation) unless otherwise indicated.

**Table 2.** Group Differences Between Aβ+ versus Aβ- on C3 at Screening Visit 1

		n=3163	n=1323	Unadjusted		Covariate-Adjusted	
		Aβ- M (SD)	Aβ+ M (SD)	Cohen’s d [95% C.I.]	p-value	Cohen’s d [95% C.I.]	p-value
	PACC	0.18 (2.45)	−0.43 (2.68)	−0.32[−0.41,−0.23]	<0.001	−0.18 [−0.25, −0.12]	<0.001
	C3 (LDI, FNMT, OCL)	0.04 (0.65)	−0.07(0.68)	−0.22 [−0.31,−0.13]	<0.001	−0.11 [−0.17, −0.04]	<0.001
BPS-O	LDI	0.41 (0.20)	0.39 (0.21)	−0.14 [−0.23, −0.05]	0.002	−0.07 [−0.14 −0.01]	0.033
FNAME	FNLT	3.76 (2.24)	3.71 (2.27)	−0.03[−0.12, 0.06]	0.526	0.03 [−0.04, 0.09]	0.402
	FNMT	8.17(1.92)	8.01 (2.04)	−0.11[−0.20, −0.02]	0.017	−0.03 [−0.10, 0.03]	0.332
	FSBT	10.48 (1.71)	10.44(1.75)	−0.03 [−0.12, 0.06]	0.526	0.03 [−0.03, 0.1]	0.330
CBB	DET RT	2.60 (0.10)	2.60 (0.11)	−0.03 [0.06, −0.12]	0.570	0.01 [0.08, −0.05]	0.686
	IDN RT	2.78(0.08)	2.77(0.08)	−0.04 [0.13, −0.04]	0.332	−0.06 [0.13, −0.00]	0.055
	IDN Acc	1.43(0.15)	1.40(0.16)	−0.25[−0.34,-0.16]	<0.0001	−0.14[−0.21, −0.08]	<0.001
	ONB RT	2.96(0.09)	2.96 (0.10)	−0.09[−0.01, −0.18]	0.037	−0.03[−0.04, −0.09]	0.384
	ONB Acc	1.38 (0.16)	1.35(0.17)	−0.23 [−0.32, −0.15]	<0.0001	−0.13[−0.19, −0.06]	<0.001
	OCL Acc	0.97(0.12)	0.95 (0.12)	−0.18[−0.26, −0.09]	<0.001	−0.09[−0.16, −0.03]	0.005

Note. M=mean, SD=standard deviation; PACC=Preclinical Alzheimer’s Cognitive Composite; C3= Computerized Cognitive Composite; BPS-O= Behavioral Pattern Separation Task-Object; LDI=Lure Discrimination Index; FNAME=Face-Name Associative Memory Exam; FNLT=1st letter Name Recall; FNMT=Face-Name Matching; FSBT=Facial Recognition; CBB=Cogstate Brief Battery; RT=reaction time; Acc=Accuracy; DET=Detection; IDN=Identification; ONB=One-Back Test; OCL=One-Card Learning.

**Table 3.** Pearson correlation coefficients (r) Among C3 Components and Demographics

		Age	Education	PACC	MMSE	FCSRT	Logical Memory	DSST
	C3 (LDI, FNMT, OCL)	−0.29	0.12	0.39	0.20	0.27	0.27	0.25
BPS-O	LDI	−0.15	0.05	0.21	0.08	0.14	0.17	0.14
FNAMF	FNLT	−0.20	0.02	0.34	0.17	0.29	0.23	0.17
	FNMT	−0.22	0.06	0.23	0.15	0.20	0.2	0.16
	FSBT	−0.22	0.06	0.27	0.13	0.20	0.13	0.22
CBB	DFTRT	−0.13	0.07	0.19	0.08	0.08	0.05	0.26
	IDN RT	−0.11	0.04	0.23	0.09	0.13	0.05	0.31
	IDN Acc	−0.17	0.01	0.12	0.07	0.09	0.06	0.09
	ONB RT	−0.16	−0.04	0.29	0.11	0.16	0.09	0.37
	ONB Acc	−0.19	0.06	0.21	0.11	0.14	0.12	0.17
	OCL Acc	−0.16	0.13	0.25	0.14	0.18	0.17	0.15

Note. Higher value represents better performance. PACC=Preclinical Alzheimer Cognitive Composite; C3= Computerized Cognitive Composite; BPS-O= Behavioral Pattern Separation Task-Object; LDI=Lure Discrimination Index; FNAME=Face-Name Associative Memory Exam; FNLT=1st letter Name Recall; FNMT=Face-Name Matching; FSBT=Facial Recognition; CBB=Cogstate Brief Battery; RT=reaction time; Acc=Accuracy; DET=Detection; IDN=Identification; ONB=One-Back Test; OCL=One-Card Learning; FCSRT=Free and Cued Selective Reminding Test; DSST=Digit Symbol Substitution Test
