# Supplementary material for: The Computerized Cognitive Composite (C3) in A4, an Alzheimer’s Disease Secondary Prevention Trial
Source: J Prev Alzheimers Dis. Author manuscript; Available in PMC 2021 Jan 1. (PMC7755110; doi:10.14283/jpad.2020.38)
Supplement: Supplementary Material [file NIHMS1646973-supplement-Supplementary_Material.docx]

SUPPLEMENTARY MATERIAL

Supplementary Table 1

Variable Definitions and Completion and Performance Checks for the C3

| **Cognitive Test** | **Main Outcome** | **Definitions** | **Completion Check** | **Performance Check** |
| --- | --- | --- | --- | --- |
| CBB DET | Speed of performance; mean of the log_10_ transformed RT for correct responses (lmn) | Detection | ≥75% of 35 trials | Accuracy ≥ 70% |
| CBB IDN | Speed of performance; mean of the log_10_ transformed RT for correct responses (lmn)  Also, accuracy of performance arcsine square root proportion correct (Acc). (secondary outcome) | Identification | ≥75% of 30 trials | Accuracy ≥ 70% |
| CBB OCL | Accuracy of performance; arcsine square root proportion correct (Acc) | One Card Learning | ≥75% of 80 trials | Accuracy ≥ 40% |
| CBB ONB | Speed of performance; mean of the log_10_ transformed RT for correct responses (lmn)  Accuracy of performance; arcsine square root proportion correct (Acc) | One Back | ≥75% of 31 trials | Accuracy ≥ 50% |
| FNAME (FNFT, FNLT, FNMT, FSBT) | FNLT-accuracy of performance (Acc)  FNMT-accuracy of performance (Acc)  FSBT-accuracy of performance (Acc) | FNFT- Face-Naming Learning  FNLT- 1^st^ Letter Name Recall  FNMT- Face-Naming Matching  FSBT-Face Recognition | 100% of 12 trials in the learning phase (FNFT)  ≥75% of 12 trials (a.k.a. at least 9 out of 12 on FNMT, FNLT and FSBT) | Accuracy ≥ 33% for FNMT  Accuracy ≥ 33% for FSBT  Accuracy n/a for FNFT and FNLT |
| BPSO (BPET, BPXT) | BPXT: The probability of calling a Distractor/Lure stimulus "Similar" minus the probability of calling a New stimulus "Similar". | BPET-object learning phase  BPXT-Pattern Separation Metric | 100% of 40 trials in the learning phase (BPET)  ≥75% of 60 trials in the recall phase (BPXT) | ≥50% Raw Accuracy on the BPXT (given random pressing can result in raw accuracy of 35% or 40%) |

NOTE. CBB-Cogstate Brief Battery, FNAME- Face Name Associative Memory Exam, BPS-O- Behavioral Pattern Separation Test-Object; RT=reaction time; In the current CBB version of the BPS-O objects from the encoding phase are presented in the recognition phase as targets and lures.

Supplementary Table 2

Linear models comparing cognitive performance between Aβ+ vs. Aβ-

|  | Dependent Variable | Independent Variables | R^2^ | Beta | F | df |
| --- | --- | --- | --- | --- | --- | --- |
| Model A | Aβ+/ Aβ- |  | 0.038 |  | 33.30** | 5, 4255 |
|  |  | PACC |  | -0.01** |  |  |
|  |  | C3 |  | -0.02** |  |  |
|  |  | Constant |  | 1.10** |  |  |
| Model B | Aβ+/ Aβ- |  | 0.029 |  | 26.50** | 5, 4386 |
|  |  | CBB IDN RT |  | 0.02 |  |  |
|  |  | CBB IDN Acc |  | -0.13** |  |  |
|  |  | Constant |  | 1.23** |  |  |
| Model C | Aβ+/ Aβ- |  | 0.024 |  | 22.00** | 5, 4277 |
|  |  | CBB OCL |  | -0.10** |  |  |
|  |  | BPS-O LDI |  | -0.03** |  |  |
|  |  | Constant |  | 1.21** |  |  |

NOTE: Age, sex, and education are included as covariates in all models (not shown). **p<0.01, PACC=Preclinical Alzheimer Cognitive Composite; C3= Computerized Cognitive Composite; BPS-O= Behavioral Pattern Separation Task-Object; LDI=Lure Discrimination Index; CBB=Cogstate Brief Battery; IDN=Identification; RT=reaction time; Acc=Accuracy
